# Biochemical Effects of Xylazine, Propofol, and Ketamine in West African Dwarf Goats

**DOI:** 10.1155/2014/758581

**Published:** 2014-09-11

**Authors:** Ukwueze Celestine Okwudili, Eze Athanasius Chinedu, Ona Jonas Anayo

**Affiliations:** ^1^Department of Veterinary Surgery and Theriogenology, College of Veterinary Medicine, Michael Okpara University of Agriculture, Umudike, PMB 7267 Umuahia, Abia State, Nigeria; ^2^Department of Veterinary Surgery and Radiology, Faculty of Veterinary Medicine, University of Nigeria, Nsukka, Nigeria

## Abstract

Anaesthesia was induced in West African Dwarf (WAD) goats using different combinations of propofol (P), xylazine (X), and ketamine (K), and the biochemical effect of the drugs determined. Twenty male (WAD) goats were randomly assigned to five treatment groups viz. Control (C) (2.5 mL IV normal saline); group K + X (5 mg/kg IV ketamine + 0.05 mg/kg IV xylazine), group P + X (5 mg/kg IV propofol + 0.05 mg/kg IV xylazine), group P + K (propofol 5 mg/kg IV + ketamine 5 mg/kg IV), and group P + K + X (propofol 2.5 mg/kg IV + ketamine 2.5 mg/kg IV + xylazine 0.05 mg/kg IV), respectively. There was increase (*P* < 0.05) in blood glucose in K + X, P + X and P + K + X. The serum cortisol level increased (*P* < 0.05) in all groups except in P + X. ALT value increased (*P* < 0.05) in K + X, P + K, and P + K + X. BUN increased (*P* < 0.05) in K + X but decreased (*P* < 0.05) in P + K + X. There was no significant variation (*P* > 0.05) in serum creatinine. These biochemical changes were transient. P + K + X would be the best drug combinations considering the biochemical parameter measured. However, data on blood glucose, ALT, BUN, and cortisol levels in an anaesthsized goat should be interpreted with caution in order to avoid erroneous interpretation in these animals.

## 1. Introduction

In Southeastern part of Nigeria, West African Dwarf (WAD) goats are the most common type of goat rear for economic purposes, hence necessitating serious attention on the health status of the animal. Anaesthetic/sedatives play significant role in both human and animal during surgery especially in painful procedures.

Propofol is a phenolic compound that has good quality anaesthesia, rapid onset, and short duration of action with rapid recoveries [1]. It lacks analgesic property and is combined with xylazine, an alpha-2-adrenoceptor agonist which has analgesic, sedative, and muscle relaxant effect, for painful procedure. Xylazine may produce cardiovascular and respiratory depression, temporal hyperglycemia in goats and cattle [2], and diuresis [3]. Ketamine is a dissociative anaesthetic agent that has profound analgesia and produces stable hemodynamic effects during anaesthesia [4].

Blood glucose is stable in ruminant most of the time because instead of being directly absorbed from the gut it is mainly derived by gluconeogenesis [5]. However, Eriksson and Teravainen [5] reported decrease in blood glucose concentration, following morning feeding of hay after which prefeeding level was restored within 2.5 hours.

Cortisol secretion increases in response to any stress in the body such as illness, trauma, surgery, temperature extremes, and transportation [6, 7]. Cortisol may improve the stress response by energy mobilization and behavioral changes [8]. The anaesthetic drugs can inhibit the release of cortisol and decrease the cortisol level in the blood [9, 10].

Alanine aminotransferase (ALT) is a liver specific hepatocellular enzyme in dog, cat, primate, and mice but not of mature horses, sheep, pigs, or cattle, as their livers do not contain significant level [11].

Blood urea nitrogen (BUN) levels may be significantly altered by protein catabolism, protein synthesis, or both [12]. Creatinine excretion is constant in the absence of disease and less affected by change in diet [13, 14].

The objective was to determine the possible biochemical effects of xylazine, propofol, and ketamine in WAD goats, ascertain the level of deviation from normal biochemical parameters of the animal, and establish biochemical profile of anaesthetized WAD goats in order to avoid erroneous interpretations when a normal blood test is analyzed in such animals.

## 2. Material and Methods

The university veterinary medical ethical committee approved this study. Twenty healthy male goats aged between 7 and 9 months were used for the study. The goats were randomly divided into four goats per a pen and kept at normal environmental temperature and natural light/darkness daily cycle. They were fed with freshly cut grasses and allowed to graze in the field under controlled tittering. Water was also served ad libitum. Clinical examination and physiological parameters such as heart rate, rectal temperature, and respiratory rate were taken regularly throughout the period of acclimatization which lasted for 21 days.

Animals were randomly assigned into five treatment groups. Group C was treated with (2.5 mL IV) normal saline. Group K + X was treated with ketamine (Laborate Pharmaceutical, India) given at 5 mg/kg with xylazine (Kepro Holland) at 0.05 mg/kg. Group P + X was treated with propofol (Pofol Popular Infusion, Ltd., Tongi, Bangladesh) given at 5 mg/kg with xylazine at 0.05 mg/kg. Group K + P was treated with propofol at the dosage of 5 mg/kg with ketamine at 5 mg/kg, while group P + K + X was treated with propofol at 2.5 mg/kg and ketamine at 2.5 mg/kg with xylazine at 0.05 mg/kg. The drugs were administered intravenously (IV) through the jugular vein. Blood samples were collected before anaesthesia at time (*t*_0_) and then at 30 min following induction and 2 hours and 24 hours after anaesthetic recovery. About 2.5 mL of blood collected was placed into non-EDTA test tube and allowed to clot and serum harvested for biochemistry. Serum cortisol levels in the experimental goat were carried out using cortisol Elisa kit with serial number E1A-1887 (DRG Diagnostic, DRG instrument GMBH, Murbarg, Germany). The procedure was carried out according to the company's specifications (DRG diagnostic user's manual, 2006). Serum ALT, BUN, and creatinine were measured by autoanalyser (BT-3000 Biotecnica, Italy). The blood glucose concentrations were measured using a hand-held glucose meter (Accu-Chek, Roche Diagnostics Auckland, New Zealand). The values obtained were expressed in mg/dL of the blood. The experiments were conducted consistently in the morning, from 7 a.m. to 11 a.m. GMT, daily.

## 3. Statistical Analysis

Data were expressed as mean ± standard error of four goats in each group. The means were compared using analysis of variance (ANOVA). A probability value less than or equal to 0.05 (*P* ≤ 0.05) was considered significant in all cases.

## 4. Result

Blood glucose at different anaesthetic periods in different groups is shown in Table 1. The values significantly increased (*P* < 0.05) at 30 min after anaesthetic induction in all the groups when compared to the preinduction values and control group. Significant increase (*P* < 0.05) was also recorded at 2 hours after anaesthetic recovery in groups K + X, P + X, and P + K + X and 24 hours after recovery in group K + X. The significant increase (*P* < 0.05) was highest in groups K + X and P + X at 30 min after anaesthetic induction when compared to other treated groups while at 2 hours after recovery it was significantly higher (*P* < 0.05) in groups K + X and P + K + X when compared to the other groups.

The serum cortisol level (Table 1) significantly decreased (*P* < 0.05) at 30 min after induction in group P + X only. However, significant (*P* < 0.05) increase at 2 hours after anaesthetic recovery was observed in groups K + X, P + K, and P + K + X. This increase remained significant at 24 hours in group P + X when compared to preinduction values and control group. The significant increase (*P* < 0.05) was higher in groups K + X and P + K + X at 2 hours after recovery when compared to other treated groups.

Serum ALT levels (*P* < 0.05) increased at 2 hours in group K + X and at 24 hours in groups P + K and P + K + X after recovery, respectively.

BUN levels increased (*P* < 0.05) in group K + X but significantly decreased (*P* < 0.05) in group P + K + X at 30 minutes after induction.

There was no significant variation (*P* > 0.05) in creatinine level in all the groups (Table 1).

## 5. Discussion

The results of this experiment showed that blood glucose level significantly increased (*P* < 0.05) in all groups at 30 minutes after induction. This may be regarded as a stress response [15]. However, it might have been probably caused by hyperglycemic effect of ketamine and xylazine [16, 17]. Xylazine has been reported to cause temporal hyperglycaemia in cattle and goats [2]. The elevation in the blood glucose may be a transient effect of the anaesthetics because the blood glucose values returned to the preinduction values at 24 hours after recovery in all groups except in group K + X. It may be as a result of hyperglycemic effect of ketamine and xylazine.

Serum cortisol level was significantly low in group P + X at 30 minutes after induction which may have resulted from the suppression of production and release of cortisol in the blood. This can be attributed to the suppressive and inhibitory action of propofol and xylazine on the adrenal steroidogenesis [18, 19]. Sharif and Abouazra [17] reported that pretreatment of goat with xylazine suppressed cortisol concentration induced by transportation. Our results show that the administration of P + X inhibited the corticoadrenal function in WAD goats, denoted by a decrease of serum glucocorticoid levels. However there was surge in the cortisol concentration at 2 hours after recovery in groups K + X, P + K, and P + K + X when the effect of the anaesthetic must have waned [20]. The elevation in the serum cortisol may be due to effect of ketamine in the combinations used. The result is in accordance with previous studies on ketamine anaesthesia [21, 22]. Cortisol release may have been triggered by the sympathomimetic effect of ketamine [22, 23]. Also ketamine may exert its antagonistic effect on the N-methyl D-aspartate (NMDA) receptors which are involved in the physiologic pulsatile regulation of hormone release from the hypothalamopituitary axis [22].

An increase in serum ALT was observed in groups K + X, P + K, and P + K + X after recovery. A similar increase in serum ALT concentration following the administration of ketamine-xylazine in rabbit has been reported [24]. Xylazine is a commonly used sedative in ruminants but there are concerns about the threat of hypoxaemia associated with its use in small ruminants [25]. It is possible that hypotension in combination with hypoxemia [24, 26, 27] may have caused the release of these enzymes from the heart muscles or liver.

Decreases in the serum BUN levels were observed following the administration of P + K + X and P + X, respectively. The observed decrease may be as a result of increase in renal function in response to the elimination of these drugs. However, there was no significant variation in creatinine concentration.

An increase in serum BUN levels was observed at 30 min after induction following the administration of K + X. The increase in serum BUN levels has been reported after K + X administration in rabbit [24, 26]. Ketamine can reduce renal cortical blood flow and urine output [28] and, hence, decreases glomerular filtration rate and increases plasma BUN and creatinine levels [24].

## 6. Conclusion

It may be concluded that the use of K + X may lead to temporal hyperglycemia. The changes in serum BUN, ALT, and creatinine observed after induction of the anaesthetics might be related to possible mild and transient effect of the anaesthetics on the renal function rather than pathological conditions. However, P + K + X would be the best drug combinations taking into account biochemical parameter measured. Nevertheless, anerroneous interpretation may arise from data on blood glucose, ALT, BUN, and cortisol level in an anaesthetized goat when a normal blood test is done in such animal due to the effect of anaesthetics on the parameters. Therefore, such data should be interpreted with caution or the blood test should be done after 24 hours after recovery.

## Figures and Tables

**Table 1:** Mean (±SEM) of different biochemical parameters in goats following intravenous administration of different anaesthetic combinations.

Parameter	C	K + X	P + X	P + K	P + K + X
Blood glucose (mg/dL)					
0 min	58.06 ± 0.62	57.52 ± 0.91	57.55 ± 1.01	58.10 ± 1.24	58.10 ± 1.24
30 min	60.6 ± 1.93	99.68 ± 4.01^∗b^	81.20 ± 1.37^∗a^	106.73 ± 3.73^∗b^	79.13 ± 4.20^∗a^
2 hr	60.75 ± 3.21	95.40 ± 3.40^∗b^	67.10 ± 3.38^∗a^	57.75 ± 3.30	86.11 ± 3.65^∗b^
24 hr	59.66 ± 1.64	78.92 ± 3.63^∗a^	57.05 ± 2.30	61.12 ± 4.20	58.85 ± 1.65
Cortisol (ng/mL)					
0 min	33.91 ± 1.38	34.35 ± 2.87	34.08 ± 3.12	31.61 ± 6.17	34.15 ± 5.43
30 min	32.25 ± 4.25	26.31 ± 8.94	12.93 ± 3.27^∗a^	21.28 ± 5.26	19.25 ± 4.25
2 hr	32.83 ± 6.31	102.11 ± 15.99^∗a^	24.78 ± 5.59	61.72 ± 7.57^*^	79.25 ± 11.58^∗a^
24 hr	35.01 ± 5.36	57.20 ± 14.68	63.46 ± 7.33^∗a^	29.54 ± 8.55	28.72 ± 7.53
ALT (iu/L)					
0 min	13.90 ± 0.90	15.24 ± 0.57	14.29 ± 0.43	14.82 ± 0.55	15.38 ± 0.72
30 min	14.05 ± 0.42	14.47 ± 1.05	14.36 ± 0.26	13.40 ± 1.38	13.40 ± 1.41
2 hr	14.16 ± 0.59	17.40 ± 1.21^∗a^	12.28 ± 1.68	13.47 ± 0.93	15.29 ± 0.27
24 hr	13.98 ± 0.29	13.50 ± 1.96	13.52 ± 2.48	20.00 ± 0.51^∗a^	24.19 ± 1.61^∗a^
Urea (mg/dL)					
0 min	32.73 ± 1.05	35.63 ± 1.72	36.13 ± 1.95	31.31 ± 1.95	33.13 ± 2.73
30 min	36.14 ± 3.93	43.86 ± 1.93^∗b^	32.72 ± 1.95	35.15 ± 1.74	21.90 ± 1.66^∗a^
2 hr	38.24 ± 3.96	38.37 ± 5.00	38.12 ± 2.09	33.97 ± 1.42	35.61 ± 1.61
24 hr	35.52 ± 2.13	30.84 ± 2.09	34.66 ± 1.73	32.01 ± 2.45	36.28 ± 2.13
Creatinine (mg/dL)					
0 min	1.54 ± 0.14	1.72 ± 0.05	1.43 ± 0.96	1.72 ± 0.09	1.68 ± 0.11
30 min	1.59 ± 0.04	1.55 ± 0.02	1.60 ± 0.06	1.60 ± 0.04	1.59 ± 0.06
2 hr	1.60 ± 0.03	1.72 ± 0.05	1.59 ± 0.12	1.65 ± 0.07	1.63 ± 0.03
24 hr	1.46 ± 0.02	1.53 ± 0.04	1.58 ± 0.07	1.57 ± 0.05	1.61 ± 0.08

^*^Data in the same column differ significantly (*P* < 0.05) from the preanaesthetic value (time 0).

^a,b^Results with different superscripts within row are significantly (*P* < 0.05) different.

C: control group, K + X: ketamine + xylazine, P + X: propofol + xylazine, P + K: propofol + ketamine, and P + K + X: propofol + ketamine + xylazine.
